# HAS-BLED vs. ORBIT scores in anticoagulated patients with atrial fibrillation: A systematic review and meta-analysis

**DOI:** 10.3389/fcvm.2022.1042763

**Published:** 2023-01-05

**Authors:** Xuyang Liu, Shengnan Wang, Wenfeng He, Linjuan Guo

**Affiliations:** ^1^Department of Cardiology, Affiliated Hospital of Jinggangshan University, Jinggangshan University, Ji’an, Jiangxi, China; ^2^Department of Medical Genetics, The Second Affiliated Hospital of Nanchang University, Nanchang, Jiangxi, China; ^3^Department of Cardiology, Jiangxi Provincial People’s Hospital Affiliated to Nanchang University, Nanchang, Jiangxi, China

**Keywords:** atrial fibrillation, ORBIT, HAS-BLED, bleeding risk, review

## Abstract

**Background:**

The 2021 UK National Institute for Health and Care Excellence guidelines tend to recommend the ORBIT score for predicting bleeding risk in patients with atrial fibrillation (AF) with anticoagulants. Herein, we comprehensively re-assessed the predicted abilities of the HAS-BLED vs. ORBIT score since several newly published data showed different findings.

**Methods:**

We comprehensively searched the PubMed electronic database until December 2021 to identify relevant studies reporting the ORBIT vs. HAS-BLED scores in anticoagulated patients with AF. Their predicted abilities were assessed using the C-index, reclassification, and calibration analysis.

**Results:**

Finally, 17 studies were included in this review. In the pooled analysis, the ORBIT score had a C-index of 0.63 (0.60–0.66), 0.59 (0.53–0.66), and 0.57 (0.48–0.67) for major bleeding, any clinically relevant bleeding, and intracranial bleeding, respectively, while the HAS-BLED score had a C-index of 0.61 (0.59–0.63), 0.59 (0.56–0.63), and 0.57 (0.51–0.64) for major bleeding, any clinically relevant bleeding, and intracranial bleeding, respectively. There were no statistical differences in the accuracy of predicting these bleeding events between the two scoring systems. For the outcome of major bleeding, the subgroup analyses based on vitamin K antagonists vs. direct oral anticoagulants suggested no differences in the discrimination ability between the HAS-BLED and ORBIT scores. Reclassification and calibration analyses of HAS-BLED vs. ORBIT should be further assessed due to the limited and conflicting data.

**Conclusion:**

Our current findings suggested that the HAS-BLED and ORBIT scores at least had similar predictive abilities for major bleeding risk in anticoagulated (vitamin K antagonists or direct oral anticoagulants) patients with AF, supporting the use of the HAS-BLED score in clinical practice.

## Introduction

Atrial fibrillation (AF) is the most common cardiac arrhythmia, the leading cause of cardiovascular diseases and death worldwide (1). Generally, the most worrisome complication of AF is cardiac stroke. Effective stroke prevention requires the use of oral anticoagulants, including vitamin K antagonists (VKAs) or direct oral anticoagulants (DOACs) (2). However, bleeding events will occur after receiving anticoagulation therapy, and it is very important to accurately assess the risk of embolism and bleeding in clinical practice. Over the past few decades, various bleeding risk scores have been proposed (3–5). Among these proposed bleeding risk scores, the HAS-BLED score (hypertension [H, 1 point], abnormal liver/renal function [A, 1 point each], stroke [S, 1 point], bleeding history or predisposition [B, 1 point], labile international normalized ratio [L, 1 point], elderly [E, 1 point], and drugs/alcohol concomitantly [D, 1 point each]) have become increasingly popular in the clinical settings (6, 7). Patients with HAS-BLED < 3 are divided into the low-risk group, while those with HAS-BLED ≥ 3 are divided into the high-risk group (2, 8).

In 2015, O’Brien et al. (9) derived and validated another bleeding risk score, ORBIT, which consists of 1 point for age ≥ 75 years, 2 points for reduced hemoglobin/hematocrit/history of anemia, 2 points for bleeding history, and 1 point for impaired renal function (<60 mL/min/1.73 m^2^). The previous meta-analysis comparing the HAS-BLED score with the ORBIT score showed that the HAS-BLED score was no better than the ORBIT score in predicting major bleeding events in anticoagulated patients with AF (10). However, this meta-analysis (10) did not compare the predictive power of the ORBIT and HAS-BLED scores for bleeding risk in different oral anticoagulant use statuses. Given recently updated research comparing the ORBIT and HAS-BLED scores, whether the ORBIT or HAS-BLED score has better predictive power for bleeding in patients with AF remains controversial. In addition, the 2021 UK National Institute for Health and Care Excellence guidelines tend to recommend the ORBIT score for predicting bleeding risk in patients with AF with anticoagulants (11). Therefore, we performed a systematic review and meta-analysis aiming to re-assess the diagnostic accuracy of the HAS-BLED vs. ORBIT scores for predicting bleeding risks in anticoagulated (VKAs or DOACs) patients with AF.

## Methods

### Literature search

We comprehensively searched the PubMed electronic database until December 2021 to identify relevant literature reporting the ORBIT vs. HAS-BLED scores in anticoagulated patients with AF. The following keywords in the search strategies were used: (1) atrial fibrillation AND (2) vitamin K antagonists OR warfarin OR coumadin OR phenprocoumon OR acenocoumarol OR indandione OR fluindione OR phenindione OR anisindione OR non-vitamin K antagonists OR direct oral anticoagulants OR dabigatran OR rivaroxaban OR apixaban OR edoxaban AND (3) HAS-BLED. We did not search “ORBIT” in this study. In addition, we checked previous reviews for additional studies (4, 5, 10, 12). Studies published in English were included in this study.

### Eligibility criteria

We included the studies if they met the inclusion criteria: (1) adult non-valvular patients with AF treated with VKAs or DOACs; (2) studies reported the diagnostic performance of the ORBIT vs. HAS-BLED scores; (c) major bleeding and any other bleeding events, such as any clinically relevant bleeding, any bleeding, intracranial bleeding, and gastrointestinal bleeding; (d) at least one of the following data were reported: C-index, net reclassification improvement (NRI) and integrated discrimination improvement [IDI] values, and calibration data. We excluded studies with insufficient data, such as reviews, case reports, comments, editorials, letters, or abstracts.

### Study selection and data extraction

Two authors independently assessed the relevant studies based on the predetermined criteria. We included the qualified articles after the title/abstract screenings and the full-text screenings. Disagreements were resolved through discussion or consultation with a third reviewer. Data were abstracted from the included studies. We abstracted the following data: author, year of publication, study type, data source, baseline patient characteristics (age, sex ratio, sample size, and type of anticoagulants), study outcomes, and follow-up time.

### Quality assessment

The quality assessment was performed using the prediction model risk of bias assessment tool (PROBAST),^1^ consisting of four domains including participants, predictors, outcomes, and analysis.

### Statistical analyses

The consistency of the included studies was assessed through the Cochrane *Q*-test and *I*^2^ index. Significant heterogeneity was considered if the *P*-value of the Cochrane *Q*-test < 0.1 or if the *I*^2^ value of >50%. In the discrimination analysis, the C-index and 95% confidence interval (CI) were abstracted from each included study and pooled by a random-effects model with an inverse variance method. The *Z*-statistic was calculated to compare the two C-indexes of the ORBIT vs. HAS-BLED models (10). For the primary major bleeding events, the subgroup analyses were conducted on the basis of VKAs vs. DOACs or available vs. unavailable labile INRs (Supplementary Table 1). We used the funnel plots to examine the publication bias, and visual inspection of asymmetry indicated a bias. In addition, we performed narrative analyses on the improvement in predictive accuracy by the reclassification analysis, including the NRI and IDI values. Calibration data represented the extent to which predicted risks correspond to observed risks.

All the statistical analyses were carried out using the Review Manager 5.4 software. A *P-*value of <0.05 indicated statistical significance.

## Results

**FIGURE 1:**
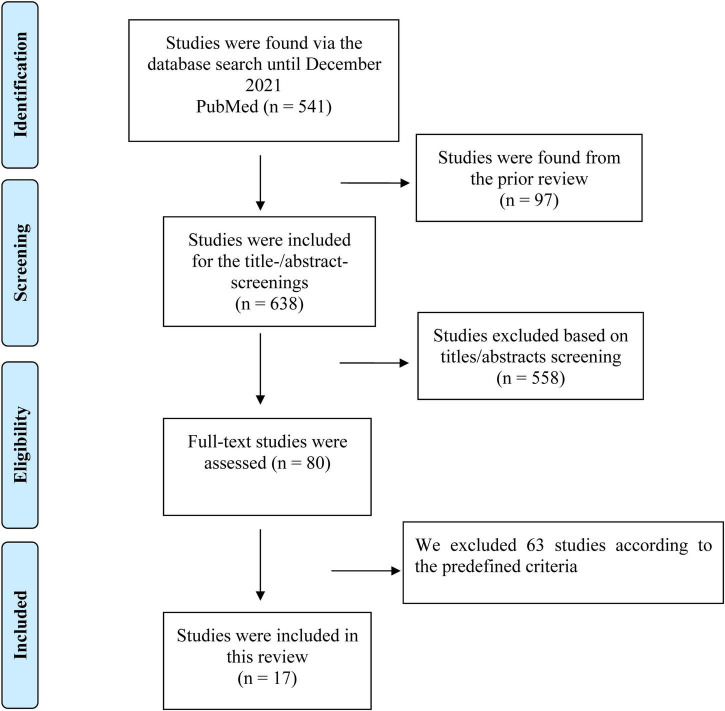
Flowchart of document retrieval and screening process of this review.

The flowchart of the document retrieval and screening process is shown in Figure 1. We initially retrieved 541 studies through an electronic search of the PubMed database. We also found additional 97 studies from the prior reviews. After the screenings of the titles and abstracts, 80 studies were assessed in full-texts, and 63 of these studies were excluded according to the predefined criteria. Finally, a total of 17 studies were included in this meta-analysis (9, 13–28).

Table 1 shows the baseline characteristics of the 17 studies included in this review. The quality assessment by the PROBAST is shown in Supplementary Table 2, suggesting that all of the included studies had a high or unclear risk of biases.

**TABLE 1:** Baseline characteristics of the included studies containing the HAS-BLED and ORBIT scores.

Included studies	Data source	Study type	Anticoagulated patients	Anticoagulated types	Study outcomes of interest	Bleeding definitions	Follow-up duration (y)	PROBAST
O’Brien et al. (9)	The ORBIT-AF registry in the USA	Prospective cohort	7,411	Dabigatran; Warfarin	Major bleeding	ISTH	2.0	Unclear
	The ROCKET-AF validation cohort	Retrospective cohort	14,264	Rivaroxaban, warfarin	Major bleeding	ISTH	1.9	
Senoo et al. (13)	The AMADEUS trial	Retrospective cohort	2,293	Warfarin	Major bleeding; Any clinically relevant bleeding	ISTH	1.18	High risk
Proietti et al. (14)	The SPORTIF III and V clinical trials	Retrospective cohort	3,551	Warfarin	Major bleeding	ISTH	1.6	Unclear
Esteve-Pastor et al. (15)	The FANTASIIA registry; Spanish	Prospective cohort	1,276	DOACs; VKAs	Major bleeding	ISTH	1.0	Unclear
Yao et al. (16)	OptumLabs Data Warehouse; US; 2010–2015	Retrospective cohort	39,539	DOACs	Major bleeding	NA	0.6	High risk
Caro Martínez et al. (17)	Three hospitals in Spain	Retrospective cohort	973	DOACs	Major bleeding; Gastrointestinal bleeding	ISTH	1.77	Unclear
Rivera-Caravaca et al. (18)	Single anticoagulation centre in a tertiary hospital in Murcia, Spain	Retrospective cohort	1,361	Acenocoumarol	Major bleeding	ISTH	6.5	Unclear
Beshir et al. (19)	University of Malaya Medical Centre and Institut Jantung Negara or the National Heart Institute of Malaysia	Retrospective cohort	1,017	Warfarin, rivaroxaban, dabigatran	Major bleeding; Any clinically relevant bleeding	ISTH	1.0	High risk
Chao et al. (20)	National Health Insurance Research Database, Taiwan	Retrospective cohort	40,450	Warfarin	Major bleeding; Intracranial bleeding	NA	4.6	Unclear
Lip et al. (21)	Three Danish nationwide databases	Retrospective cohort	57,930	DOACs	Any bleeding	ICD codes	2.5	Unclear
Proietti et al. (22)	The RE-LY trial, whole cohort	Retrospective cohort	18,113	Dabigatran; warfarin	Major bleeding; Intracranial bleeding	ISTH	2.0	Unclear
Claxton et al. (23)	The derivation (MarketScan, 2007–2014) and validation (Optum Clinformatics, 2009–2015) cohorts	Prospective cohort	81,285	DOACs; Warfarin	Major bleeding	ISTH	1.0	High risk
Rutherford et al. (24)	Norwegian Patient Registry and Norwegian Prescription Database	Retrospective cohort	21,248	DOACs	Any clinically relevant bleeding	ICD codes	0.5	High risk
Mori et al. (25)	The DIRECT registry in Japan	Prospective cohort	2,216	DOACs	Major bleeding	ISTH	0.86	High risk
Adam et al. (26)	Multicenter cohort study in Switzerland	Prospective cohort	2,147	DOACs; VKAs	Any clinically relevant bleeding	ISTH	4.4	Unclear
Watanabe et al. (27)	J-RHYTHM Registry	Prospective cohort	7,406	VKAs	Major bleeding	NA	2.0	Unclear
Proietti et al. (28)	ESC-EHRA EORP-AF General Long-Term Registry	Prospective cohort	3,018	DOACs	Major bleeding	NA	2.0	Unclear

Only analyzed in the analysis of the DOAC subgroup based on the occurrence of intracranial hemorrhage and major extracranial hemorrhage during the follow-up.

HAS-BLED, hypertension, abnormal liver/renal function, stroke, bleeding history or predisposition, labile international normalized ratio, elderly, drugs/alcohol concomitantly; ORBIT, outcomes registry for better informed treatment of atrial fibrillation; ICD, International Classification of Diseases; ISTH, International Society of Thrombosis and Haemostasis; VKAs, vitamin K antagonists; DOACs, direct oral anticoagulants; NA, not available.

### Discrimination analysis between HAS-BLED and ORBIT

**FIGURE 2:**
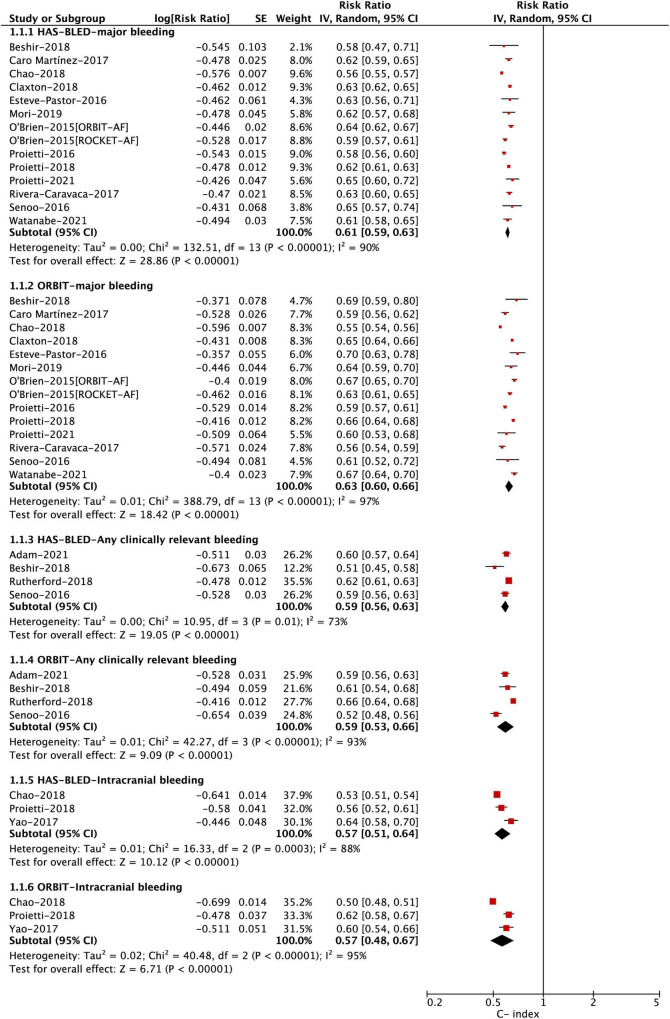
Pooled C-index for bleeding events in anticoagulated patients with atrial fibrillation.

**FIGURE 3:**
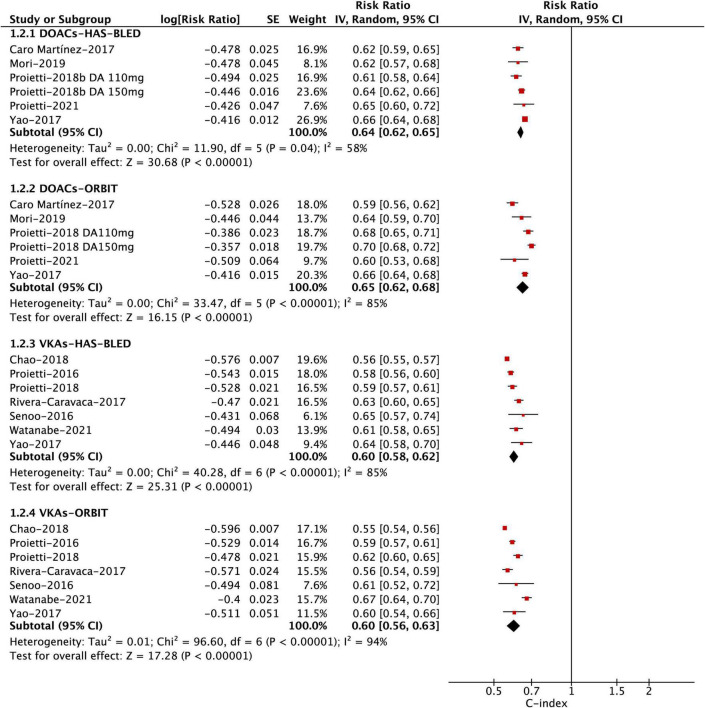
Pooled analysis of the *C*-statistics of different subgroups by using DOAC or VKA for major bleeding.

The discrimination analysis was assessed by the C-index between the HAS-BLED and ORBIT scores. In the pooled analysis shown in Figure 2 and Table 2, the ORBIT score had a C-index of 0.63 (0.60–0.66), 0.59 (0.53–0.66), and 0.57 (0.48–0.67) for major bleeding, any clinically relevant bleeding, and intracranial bleeding, respectively. Similarly, the HAS-BLED score had a C-index of 0.61 (0.59–0.63), 0.59 (0.56–0.63), and 0.57 (0.51–0.64) for major bleeding, any clinically relevant bleeding, and intracranial bleeding, respectively. The *Z*-statistics suggested that the two scoring systems had no statistical differences in the accuracy of predicting bleeding events (major bleeding, any clinically relevant bleeding, and intracranial bleeding) after anticoagulation in patients with AF. For the outcome of major bleeding, the subgroup analyses based on the OAC type suggested that there were no differences in the discrimination ability between the HAS-BLED and ORBIT scores in either the DOAC or VKA group (Figure 3 and Table 2). In addition, the subgroup analyses based on available vs. unavailable labile INRs also showed no difference.

**TABLE 2:** *C*-statistics and 95% CIs between the HAS-BLED and ORBIT scores.

	Major bleeding	Any clinically relevant bleeding	Intracranial bleeding
**Overall**			
No. of studies	14	4	3
C-statistic: HAS-BLED	0.61 (0.59–0.63)	0.59 (0.56–0.63)	0.57 (0.51–0.64)
C-statistic: ORBIT	0.63 (0.60–0.66)	0.59 (0.53–0.66)	0.57 (0.48–0.67)
**Subgroup analysis**			
**DOAC-group**			
No. of studies	6		
C-statistic: HAS-BLED	0.64 (0.62–0.65)	–	–
C-statistic: ORBIT	0.65 (0.62–0.68)	–	–
**VKA-group**			
No. of studies	7		
C-statistic: HAS-BLED	0.60 (0.58–0.62)	–	–
C-statistic: ORBIT	0.60 (0.56–0.63)	–	–

HAS-BLED, hypertension, abnormal liver/renal function, stroke, bleeding history or predisposition, labile international normalized ratio, elderly, drugs/alcohol concomitantly; ORBIT, outcomes registry for better informed treatment of atrial fibrillation; VKAs, vitamin K antagonists; DOACs, direct oral anticoagulants; CI, confidence interval.

### Reclassification analysis between HAS-BLED and ORBIT

The NRI and IDI data between the HAS-BLED and ORBIT scores are presented in Supplementary Table 3. For the primary outcome of major bleeding, only four included studies reported the NRI and IDI values between the two studied risk scores. Consistently, the HAS-BLED score in these four studies showed positive NRI and IDI values compared with the ORBIT score, although not significant in each included study. In addition, for the outcome of intracranial bleeding, Chao et al. reported that the HAS-BLED score had a significantly positive NRI value (+4.8%, *P* < 0.001) compared with the ORBIT score. No NRI and IDI data were reported about any other bleeding outcomes.

### Calibration analysis between HAS-BLED and ORBIT

Calibration data between the HAS-BLED and ORBIT scores from seven included studies are displayed in Supplementary Table 4. However, we found that their findings of this part were not consistent among the included studies. Proietti et al., Lip et al., O’Brien et al., and Watanabe et al. showed that ORBIT had a better calibration than HAS-BLED, while Proietti et al. acquired the opposite finding. Beshir et al. and Mori et al. found no difference in the calibration data between ORBIT and HAS-BLED.

### Publication bias

As shown in Supplementary Figure 1, when analyzing the C-index, we found no potential publication biases when inspecting the funnel plot.

## Discussion

The goal of anticoagulation in patients with AF is to minimize the occurrence of adverse events (especially the risk of stroke) by preventing thrombosis, while the risk of bleeding is significantly increased during anticoagulation. Therefore, the accurate prediction of patients with AF with a high bleeding risk is helpful. To date, multiple bleeding risk scores have been created for the assessment of bleeding risk in patients with AF on anticoagulation (29). Moreover, the HAS-BLED and ORBIT scores have been validated in different populations, especially patients with AF using DOACs. These two scores, especially the HAS-BLED score, are currently the most commonly used score in trials and clinical practice systems. Therefore, this study mainly conducted a meta-analysis on the predictive power of the ORBIT vs. HAS-BLED scores in patients with AF with different anticoagulants (VKAs or DOACs).

Our results showed that ORBIT was comparable to the HAS-BLED score in predicting major bleeding, any clinically relevant bleeding, and intracranial hemorrhage in patients with AF treated with anticoagulation. However, the HAS-BLED score was found to be more predictive than the ORBIT score in terms of NRI and IDI values. Because the criterion of “unstable INR” is difficult to be defined and detected, the HAS-BLED score is less suitable for patients with AF who have received anticoagulation therapy with DOACs or have not received anticoagulation therapy. By contrast, the ORBIT score no longer includes “unstable INR.” Subsequent studies comparing the ORBIT with the HAS-BLED score showed greater variability in the predictive values (13, 16, 28, 30). Some cohort studies showed that the HAS-BLED was superior to the ORBIT score in predicting bleeding risk in patients with VKAs or DOACs (28), potentially consistent with our current findings. While in patients with AF receiving VKAs, the ORBIT score was less predictive in ultimately identifying patients with truly “low risk” of major bleeding but was ultimately in identifying truly “low risk” major bleeding in patients with AF receiving VKA anticoagulation. The exclusion of unstable INR or alternative assessment of anticoagulation-related outcomes (reduced hemoglobin/hematocrit/history of anemia) may have resulted in an underestimation of bleeding risk by the ORBIT score. Interestingly, the revised ORBIT (including the unstable INR) showed better clinical utility and higher predictive power than the original ORBIT score. Senoo et al. compared the ORBIT score with TTR or not; for the ORBIT score, adding time in the therapeutic range (TTR) would result in a significant improvement in AUC (*p* = 0.002), with an NRI of 0.26 (*p* < 0.001) and IDI of 0.0065 (*p* < 0.001), compared with ORBIT score without TTR. The difference in AUC between the HAS-BLED score and the ORBIT score was also significant (*p* = 0.002), while NRI and IDI values were not evaluated (13). Proietti et al. made the same comparison, and the result was significantly raised in AUC (*p* = 0.106), with an NRI of 0.2508 (*p* = 0.0054) and IDI of 0.0023 (*p* = 0.0092) (14). Rivera-Caravaca et al. also produced similar results with an NRI of 0.1097 (*p* < 0.001) and IDI of 0.0270 (*p* < 0.001) (18). Ultimately, we believe that the ORBIT score is not significantly better than the HAS-BLED score as it is unlikely to underestimate the bleeding risk in patients anticoagulated with VKAs or DOACs.

In addition, in the subgroup analysis based on the OAC type (VKAs vs. DOACs), we found that the HAS-BLED and ORBIT scores had similar moderate abilities for predicting major bleeding. Therefore, for patients with AF treated with VKA or DOAC anticoagulation, it is still recommended to use the HAS-BLED score for bleeding risk assessment among patients with AF. Moreover, the assessment of bleeding risk is not a “static” process, and patients with AF need to be repeatedly assessed throughout the course of anticoagulation therapy. The main role of the bleeding risk score is to “mark” patients who may be at risk of bleeding for more careful evaluation and follow-up.

### Limitations

Several limitations in this meta-analysis should be noted. First, there was high heterogeneity in the C-index analysis and limiting data of the NRI and IDI values, calibration, and decision curve analysis between the two studied scores. Second, the study quality assessed by the PROBAST was relatively low for each included study, potentially limiting the reliability of our findings. Third, beyond major bleeding, the predictive ability of other bleeding events between the HAS-BLED and ORBIT scores should be further explored.

## Conclusion

The HAS-BLED and ORBIT scores had similar predictive abilities for major bleeding risk in VKA- or DOAC-treated patients with AF, supporting the recommendation of the HAS-BLED score in the AF settings.

## Data availability statement

The original contributions presented in this study are included in the article/Supplementary material, further inquiries can be directed to the corresponding authors.

## Author contributions

All authors listed have made a substantial, direct, and intellectual contribution to the work, and approved it for publication.

## Footnotes

1
www.probast.org
