# Supplementary material for: HAS-BLED vs. ORBIT scores in anticoagulated patients with atrial fibrillation: A systematic review and meta-analysis
Source: Front Cardiovasc Med. 2023 Jan 5;9:1042763. doi: 10.3389/fcvm.2022.1042763 (PMC9849745; doi:10.3389/fcvm.2022.1042763)
Supplement: Supplementary file 1 [file Data_Sheet_1.DOCX]

**Supplemental Table 1. Available or unavailable labile INRs of HAS-BLED score in the included studies**

| **Studies** | **Labile INRs** | |
| --- | --- | --- |
|  | **available** | **Unavailable** |
| O'Brien-2015  Senoo-2016  Proietti-2016  Esteve-2016  Yao-2017  Caro Martínez-2017  Rivera-Caravaca-2017  Beshir-2018  Chao-2018  Lip-2018  Proietti-2018  Claxton-2018  Rutherford-2018  Mori-2019  Adam-2021  Watanabe-2021  Proietti-2021 | √  √  √  √  √  √  √ | √  √  √  √  √  √  √  √  √  √ |

**Supplemental Table 2. The risk of bias assessment for all included studies using the PROBAST checklist**

| **Studies** |  | **Risk of Bias** |  |  | **Overall** |
| --- | --- | --- | --- | --- | --- |
|  | **Patients** | **Predictors** | **consequences** | **Analysis** |  |
| O'Brien-2015 |  |  |  |  |  |
| Senoo-2016 | Low risk | Unclear | High risk | Unclear | High risk |
| Proietti-2016 | Low risk | Unclear | Unclear | Unclear | Unclear |
| Esteve-2016 | Low risk | Unclear | High risk | Unclear | High risk |
| Yao-2017 | Low risk | Unclear | Low risk | Unclear | Unclear |
| Caro Martínez-2017 | Low risk | Unclear | Low risk | Unclear | Unclear |
| Rivera-Caravaca-2017 | Low risk | Unclear | High risk | Unclear | High risk |
| Beshir-2018 | Low risk | Unclear | Low risk | Unclear | Unclear |
| Chao-2018 | Low risk | Unclear | Low risk | Unclear | Unclear |
| Lip-2018 | Low risk | Unclear | Low risk | Unclear | Unclear |
| Proietti-2018 | Low risk | Unclear | Low risk | Unclear | Unclear |
| Claxton-2018 | Low risk | Unclear | Low risk | Unclear | Unclear |
| Rutherford-2018 | Low risk | Unclear | Unclear | Unclear | Unclear |
| Mori-2019 | Low risk | Unclear | High risk | Unclear | High risk |
| Adam-2021 | Low risk | Unclear | High risk | Unclear | High risk |
| Watanabe-2021 | Low risk | Unclear | Low risk | Unclear | Unclear |
| Proietti-2021 | Low risk | Unclear | Low risk | Unclear | Unclear |

**Supplemental Table 3. The NRI and IDI data between the HAS-BLED and ORBIT scores**

| **Studies** | **NRI values** | **IDI values** |
| --- | --- | --- |
| **Major bleeding** |  |  |
| Chao-2018 | HAS-BLED vs. ORBIT (+5.5%, P<0.001) | Not available |
| Proietti-2016 | ORBIT vs. HAS-BLED (-0.77%, P=0.392) | ORBIT vs. HAS-BLED (0%, P=0.646) |
| Rivera-Caravaca-2017 | HAS-BLED vs. ORBIT (+12.12%, P=0.007) | HAS-BLED vs. ORBIT (+2.4%, P=0.067) |
| Proietti-2021 | ORBIT vs. HAS-BLED (-11.7%, P=0.093) | ORBIT vs. HAS-BLED (-0.2%,, P=0.691) |
| **Intracranial bleeding** |  |  |
| Chao-2018 | HAS-BLED vs. ORBIT (+4.8%, P<0.001) | Not available |

**Supplemental Table 4. Calibration data between the HAS-BLED and ORBIT scores**

| Proietti-2018 | HAS-BLED overestimated the risk, ORBIT showed better calibration than HAS-BLED |
| --- | --- |
| Beshir-2018 | Adequate calibration, no difference between ORBIT and HAS-BLED |
| Lip-2018 | HAS-BLED underestimated the risk, ORBIT appeared well-calibrated up to score 4. |
| Mori-2019 | The ORBIT bleeding score showed a similar predictive performance compared with the HAS-BLED score (slope: 0.91 [95% CI 0.40, 1.43] vs 0.72 [95% CI 0.03, 1.40], intercept: 0.24 [95% CI 2.13, 2.61] vs 0.71 [95% CI 2.35, 3.76], respectively) |
| O'Brien-2015 | The ORBIT score displayed superior calibration compared with the other 2 scores, followed by HAS-BLED (worst at low risk strata) and ATRIA (not good for most risk groups) |
| Watanabe-2021 | ORBIT showed better calibration than HAS-BLED throughout all risk strata |
| Proietti-2021 | ORBIT showed poorer calibration than HAS-BLED throughout all risk strata |


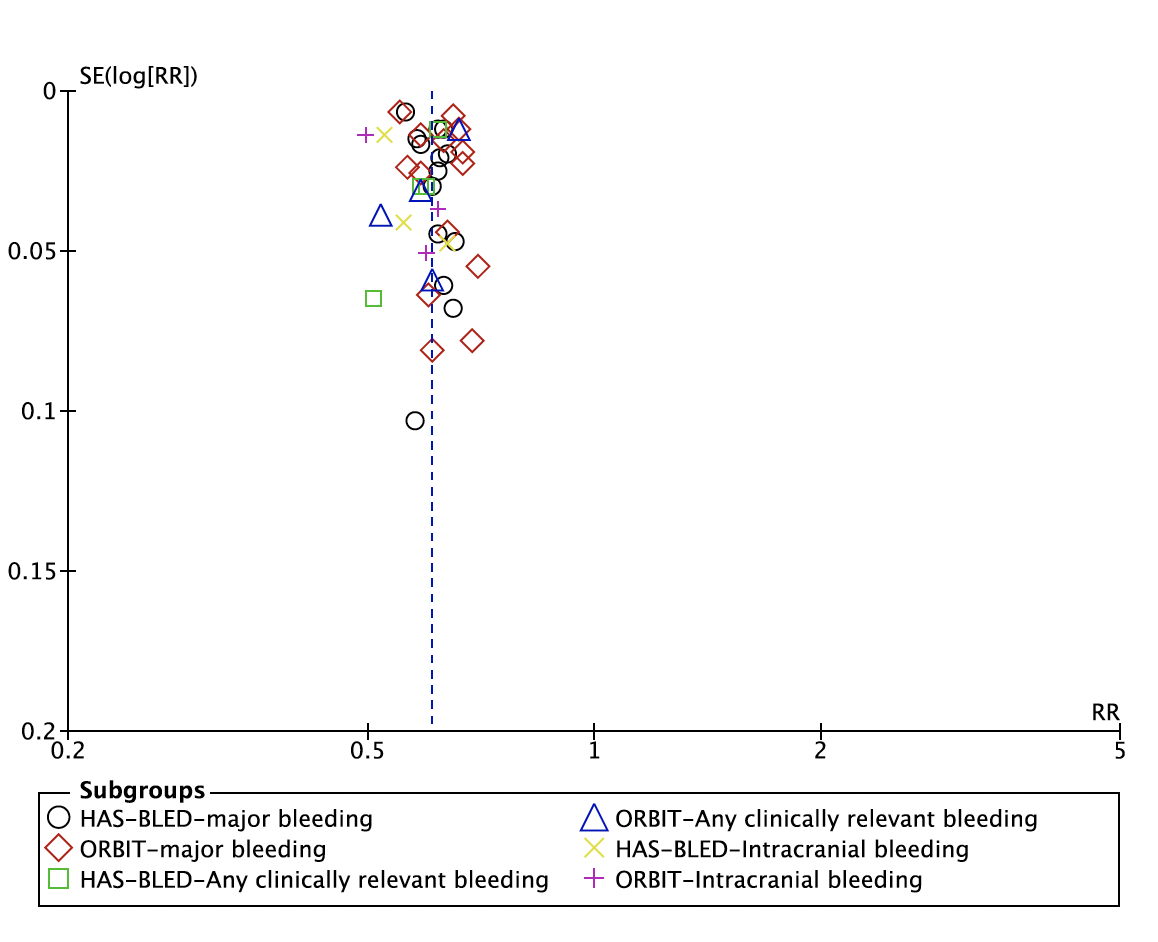


**Supplemental Figure 1. The funnel plot to examine the publication bias**
